# Supplementary material for: Seipin is required for converting nascent to mature lipid droplets
Source: eLife. 2016 Aug 26;5:e16582. doi: 10.7554/eLife.16582 (PMC5035145; doi:10.7554/eLife.16582)
Supplement: Supplementary file 1. — (A) Sequences of primers used to generate dsRNA. (B) Sequences of primers used for qPCR. (C) List of plasmids used. DOI: http://dx.doi.org/10.7554/eLife.16582.031 [file elife-16582-supp1.docx]

**Supplementary file 1A. Sequences of primers used to generate dsRNA.**

| **Gene Name** | **Gene ID** | **Forward** | **Reverse** |
| --- | --- | --- | --- |
| Seipin | CG9904-3'UTR | TAATACGACTCACTATAGGG ATGGAGGCGGAGATGATATG | TAATACGACTCACTATAGGG AGCTAATTGGCTTGCAATGG |
| Seipin | CG9904 | TAATACGACTCACTATAGGGACGCCCTGCACCTTTCC | TAATACGACTCACTATAGGGACTATGGCCGACAATACGG |
| GPAT1 | CG5508 | AACCT1GCAAATTCCGGTCT | CAGATGGCTTGTAGACCT1TG |
| GPAT4 | CG3209 | CGGCGACGCCTTCTG | CAGCCGATTGGCAAACTC |
| AGPAT2 | CG17608 | GCTCCCAAAAATGGCTTGTA | GAACGCAAAGATCAACAGCA |
| AGPAT3 | CG4729 | GAGTTTGCTGCATGGAAAG | AGTCCCAGGACGGAAAGT |
| LIPIN | CG8709 | GTGGTGCTGGGTCAGGTC | TCCCT1CTGCTGCTTGGT |
| DGAT1 | CG31991 | AACCGCAAGTCAACACAAAA | AAGCCCAAACGCAGACC |
| DGAT2 | CG1942 | CAAGTGGCTGGAGCTATTC | GGATCAGCGCCT1CTTTG |
| CCT1 | CG1049 | ACATCTATGCTCCT1CTCAAGGC | CTCTGCAGACTCTGGTAACTGC |
| ECT | CG5547 | GTC TTG TTT AGT ATG TCG TCC CC | TTG GAA TTC GCA ATA TTT TTG G |
| pBluescript |  | AATTCGATATCAAGCTTATCGAT | TAAATTGTAAGCGTTAATATTTTG |

**Supplementary file 1B. Sequences of primers used for qPCR.**

| **Gene Name** | **Gene ID** | **Forward** | **Reverse** | **For** |
| --- | --- | --- | --- | --- |
| dSeipin | CG9904 | GTTCAAAACCTGCCTGGAGA | CGATGTTCACGATCACCTTG | RNAi confirmation |
| hSeipin / BSCL2 | 26580 | CTCAGCCCTGTGCATTT | ATCACGTCCACCCTTAGT | CRISPIR KO confirmation |
| Xbp-1 | 7494 | CCTGGTTGCTGAAGAGGAGG | TGGGGAGATGTTCTGGAG | ER stress |

**Supplementary file 1C. List of plasmids used.**

| **Name** | **Insert** | **Region (aa)** | **Vector** | **Species** |
| --- | --- | --- | --- | --- |
| GFP-*LiveDrop* | dGPAT4 | 160-216 | pAGW | Drosophila |
| Cherry-*LiveDrop* | dGPAT4 | 160-216 | pACW | Drosophila |
| ss-KDEL-BFP2 | signal sequence+KDEL+BFP2 |  | pAW | Drosophila |
| cherry-dSeipin | dSeipin/CG9904 | full length (1-370) | pACW | Drosophila |
| dSeipin-cherry | dSeipin/CG9904 | full length (1-370) | pAWC | Drosophila |
| cherry-dSeipin-NT | dSeipin/CG9904 | 1-45 | pACW | Drosophila |
| cherry-dSeipin-CT | dSeipin/CG9904 | 279-370 | pACW | Drosophila |
| cherry-dSeipin-ΔN | dSeipin/CG9904 | 45-370 | pACW | Drosophila |
| cherry-dSeipin-ΔC | dSeipin/CG9904 | 1-279 | pACW | Drosophila |
| cherry-dSeipin-loop | dSeipin/CG9904 | 45-279 | pACW | Drosophila |
| cherry-Lunapark | Lunapark/CG8735 | Full length | pACW | Drosophila |
| hSeipin-GFP | human Seipin | Full length | pAWG | Drosophila |
| ss-KDEL-BFP2 | signal sequence+KDEL |  | mTagBFP2 | Mammalian |
